# Haemobilia Associated With a Postoperative Biliary Stricture

**DOI:** 10.1155/1989/13926

**Published:** 1989

**Authors:** Thomas F. Gorey, John Drumm, Patrick G. Collins

**Affiliations:** Department of Surgery Royal College of Surgeons in Ireland The Charitable Infirmery Jervisal Street Dublin 1 Ireland


*HPB Surgery* 1989, Vol. 1, pp. 229–231
Reprints available directly from the publisher
Photocopying permitted by license only

© 1989 Harwood Academic Publishers GmbH
Printed in Great Britain

### CASE REPORT
### HAEMOBILIA ASSOCIATED WITH A POSTOPERATIVE BILIARY STRICTURE

THOMAS F. GOREY*, JOHN DRUMM and PATRICK G. COLLINS

*Department of Surgery, Royal College of Surgeons in Ireland, The Charitable Infirmery, Jervisal Street, Dublin 1, Republic of Ireland*

*KEY WORDS: Haemobilia, biliary stricture*

### INTRODUCTION

Haemobilia is an unusual cause of gastrointestinal (GI) bleeding with a mortality rate in the region of 25%. Approximately 50% is due to trauma[1], and 14% to operative trauma. The source of haemobilia is often obscure and, as Sandblom pointed out, even a negligible communication between the arterial and ductal systems may give rise to intense haemobilia if coagulation is defective, bile itself being fibrinolytic. He reported that bleeding is from the liver in 50% of cases, the gallbladder in 25%, and the ducts in 25%.

### CASE REPORT

A 60-year-old female was referred seven weeks after cholecystectomy for repair of a suspected biliary stricture. She was readmitted a month after discharge with a 5-day history of jaundice and rigors. The only operative comment of note was that there has been "difficulty with accidental bleeding from the cystic artery". Attempted percutaneous transhepatic cholangiography failed; bile was aspirated but no ducts were outlined. At laparotomy there was a 6-cm diameter cavity in the right lobe of the liver with a stricture just distal to the duct bifurcation. The entire ductal system contained clot, which formed an almost perfect cast. An hepaticojejunostomy was performed and the patient made an uneventful recovery with consistent clearing of jaundice.

Three weeks later she had significant melaena with a fall in systolic pressure to 60 mmHg. Over 12 h she received eight units of blood and fresh frozen plasma, and at subsequent laparotomy the Roux loop to her duct reconstruction was full of blood. A precise bleeding point was not located and the haematologist reported thrombocytopaenia with platelet dysfunction. Over the next five days, despite multiple transfusions and platelet concentrate at the rate of 12 units per day as well as parental vitamin K, B12 and folic acid, she continued to bleed, became anuric and died.

At autopsy the Roux loop was grossly distended with blood but there was no obvious source of gastrointestinal bleeding. The liver, with the hepaticojejunostomy, and great vessels were excised *en bloc*. The coeliac axis was

* Correspondence to: Mr T. F. Gorey, MCh, FRCSI, The Charitable Infirmary, Jervis Street, Dublin 1, Republic of Ireland.

cannulated from within the aortic lumen, and hepatic angiography carried out. This showed a 2-cm diameter aneurysm of the right hepatic artery just within the liver substance (Figure 1). The specimen was dissected and the aneurysm located after a catheter was passed through the coeliac axis. On dissection there was found a 2/0 silk ligature at this site, confirmed histologically to be a false aneurysm.

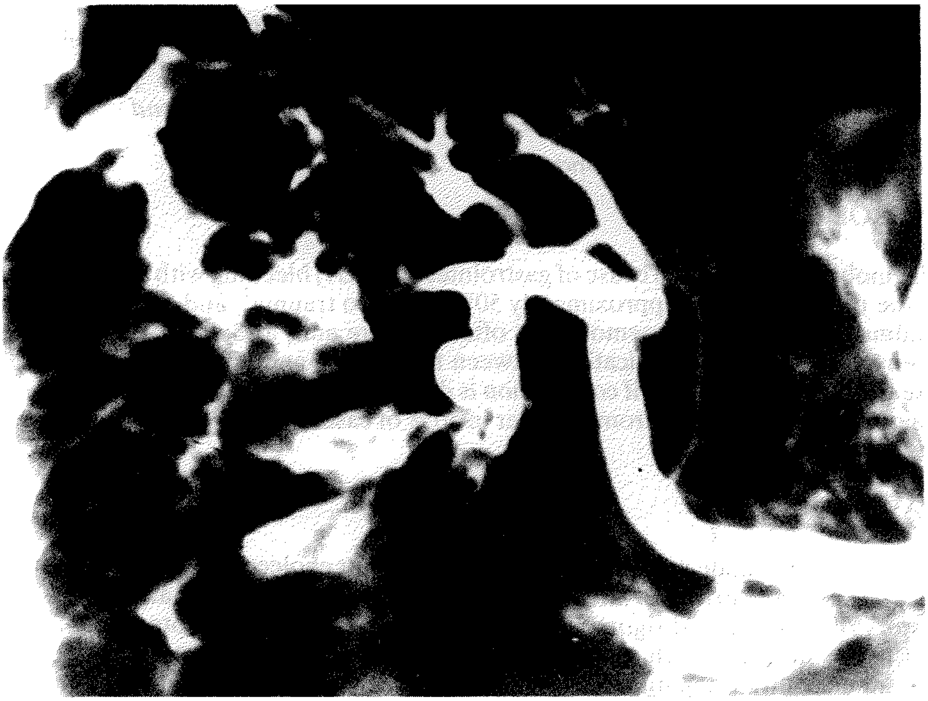

**Figure 1** An autopsy angiogram showing a 2-cm diameter right hepatic artery false aneurysm.

### DISCUSSION

In this case there was blood in the biliary tree at the corrective operation, and subsequently the jaundice was resolving. The classical triad of haemobilia is biliary colic, jaundice and GI haemorrhage, but colic may be absent in these reconstruction cases with no extrahepatic biliary tree. Diagnosis is difficult; angiography is essential and may also offer a therapeutic option by embolization.

In this patient the aneurysm was located only at autopsy after X-ray and by dissection after a catheter was passed through the coeliac axis. Treatment is either hepatic resection or hepatic artery ligation; Mays[2] reported good results for ligation, especially for hilar lesions. The incidence of haemobilia is increasing with the increase in hepatic trauma, but mortality is declining with better diagnosis and more aggressive therapy. Iatrogenic injuries may be due to traumatic instrumentation or to a false aneurysm from hepatic artery damage — the probable cause in this case. Microscopic haemobilia can also occur during peroperative cholangiography (4%).

Congential and atherosclerotic aneurysms are rare in the hepatic arterial system, and in the Far East ascaris can be a cause of "tropic haemobilia".

Biliary vascular communications are usually associated with an intrahepatic cavity, and in this case a cavity 6 cms in diameter was also responsible for the failed PTC.

In conclusion, it is emphasized that arterial damage must be considered if haemobilia is encountered during repair of a postoperative biliary stricture. It would seem prudent that all cases of haemobilia merit arteriography, as the extent of haemorrhage is otherwise impossible to determine clinically. It is further suggested that in any unexplained deaths from GI bleeding it may be worthwhile doing an autopsy angiogram to display the intrahepatic vessels.

### *References*

1. Sandblom, P. (1973) Haemobilia. *Surg. Clin. N.Am.*, **53**, 1191–1201.
2. Mays, E.T. (1972) Lobar dearterialization for exsanguinating wounds of the liver. *J. Trauma,* 12, 397-9.

*Accepted by S. Bengmark 16 February 1988*

